# Advances of Emerging Memristors for In-Memory Computing Applications

**DOI:** 10.34133/research.0916

**Published:** 2025-10-09

**Authors:** Qingxin Chen, Lin Lu, Jialin Meng, Mingsheng Xu, Tianyu Wang

**Affiliations:** ^1^ School of Integrated Circuits, Shandong University, Jinan 250100, China.; ^2^ Suzhou Research Institute of Shandong University, Suzhou 215123, China.; ^3^ State Key Laboratory of Crystal Materials, Shandong University, Jinan 250100, China.; ^4^ National Integrated Circuit Innovation Center, Shanghai 201203, China.; ^5^ Institute of Novel Semiconductor Materials, Shandong University, Jinan 250100, China.

## Abstract

The memristor, as an emerging nonvolatile device, has garnered considerable attention due to its low power consumption, high density, and multifunctionality. This review summarizes recent advances in the application of memristors for logic gates, with a focus on key breakthroughs and challenges in material design, device performance, and logic circuit implementation. It covers a variety of material systems including 2-dimensional materials, perovskite materials, and optoelectronic materials, as well as novel structures such as array architectures and wearable textile memristors, evaluating their suitability for achieving stable and efficient logic operations. In addition, the review provides a comparative analysis of different implementation strategies for basic logic, optoelectronic logic, and combinational logic, offering an in-depth discussion of their respective characteristics and advantages. This review also emphasizes the application prospects of memristor logic gates in reconfigurable computing, neuromorphic computing, and in-memory computing architectures, providing a theoretical foundation and practical support for the development of high-density integration and efficient memristor logic circuits.

## Introduction

Logic gates are the core electronic devices that realize basic logic operations and constitute the basic building blocks of modern digital circuits and computing systems. They are the key to the realization of the functions of computers, communications, and embedded systems by performing logical operations on input signals [1–3]. In traditional von Neumann architectures, energy consumption and data latency are significantly increased due to the dependence of logic operations on frequent data transfers between the central processor and memory [4–6]. The traditional logic gates based on complementary metal-oxide semiconductor (CMOS) technology are difficult to meet the increasing computational complexity and low-power requirements due to their power consumption and large integration area [7–10]. The operation principle of CMOS logic gates relies on the switching behavior of the transistors, and their dynamic power consumption increases as the transistor size is further reduced, which limits their further application in ultralarge-scale integrated circuits [11–16].

As a new type of nonvolatile device, the memristor has revolutionized logic gate design. Memristors are able to realize logic operations through the characteristic of resistive switching without the need for additional memory cells, thus breaking through the limitations of traditional CMOS logic gates [17–20]. Compared to traditional logic gates, memristor logic gates offer lower power consumption, higher integration density, and the potential to support polymorphic logic operations [21–24]. In addition, the memristors support an integrated memory-computer architecture, which enables data storage and logic operations to be realized simultaneously in the same device, thus effectively alleviating the data transfer bottleneck in the von Neumann architecture [25–28]. This advantage makes the memristor an important application prospect in logic computing, neuromorphic computing, and edge computing and provides a new way of thinking about the design of next-generation computing architectures [29,30].

**Fig. 1.**
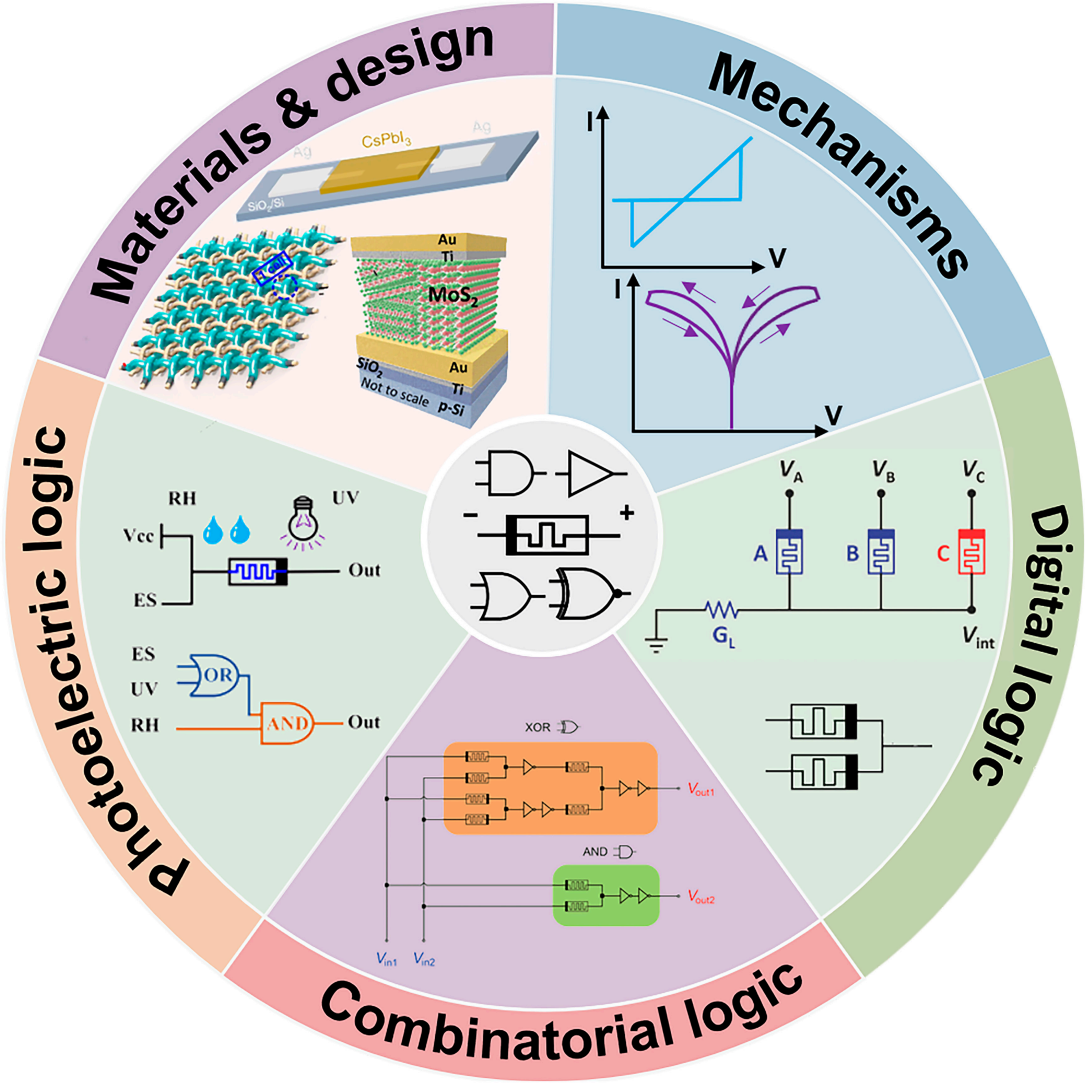
Materials and design, digital logic, combinatorial logic, and photoelectric logic [46,49,55,95,109,132]. Copyright 2017, American Chemical Society (ACS). Copyright 2020, Zhu et al. Copyright 2022, ACS. Copyright 2018, WILEY-VCH Verlag GmbH & Co. KGaA, Weinheim. Copyright 2022, Tsinghua University Press. Copyright 2024, ACS.

This review systematically summarizes various memristor material structures, the design of memristor-based logic gates, and their applications in modern computing systems. It covers key aspects such as materials, device structures, working mechanisms, performance metrics, input modalities, and logic function implementation strategies, as illustrated in Fig. 1. We focus on how memristors with different material configurations achieve fundamental Boolean logic operations through their resistive switching characteristics. The input modes of memristor logic gates not only include conventional electrical signals but also extend to optical inputs and optoelectronic hybrid stimuli [31,32]. Optical input allows memristor logic gates to perceive environmental information through light–material interactions, thereby enhancing signal processing speed [33–35]. Through diverse design architectures, memristor logic gates can not only implement basic Boolean logic but also be configured into combinational logic circuits and more advanced processing units, showing great potential for practical logic computing applications [36]. Finally, this review discusses the future prospects of memristor logic gates in efficient computing architectures, particularly in the contexts of reconfigurable computing, neuromorphic systems, and in-memory computing frameworks [37,38]. We believe that memristor logic gates will play a crucial role in next-generation high-performance computing, facilitating the transition from conventional to novel computing paradigms.

## Structure and Performance of Memristors

### Structure and working mechanism

The implementation of memristor logic gates relies on the properties of the materials used, structural design, and performance optimization. These factors collectively determine the computation speed, power consumption, and stability of the logic gates [39,40]. In memristors, an applied voltage can induce the migration of ions within the material or the redistribution of interface charges, leading to a change in the resistance state [switching between high (HRS) and low resistance (LRS) states]. This resistance switching characteristic enables memristors to perform logical operations. Among the factors affecting memristor performance, material selection plays a central role. The conductivity, ion migration behavior, and environmental adaptability of emerging materials—such as 2-dimensional materials, perovskites, and photosensitive compounds—directly influence the switching speed, power consumption, and operational reliability of the devices [40–42]. In addition to materials, device architecture is equally important. Metal–insulator–metal (MIM) structures, for instance, can enhance local electric field strength and improve the ON/OFF switching ratio. Meanwhile, fiber-based configurations offer promising opportunities for implementing memristor logic in flexible and wearable electronics [43].

**Fig. 2.**
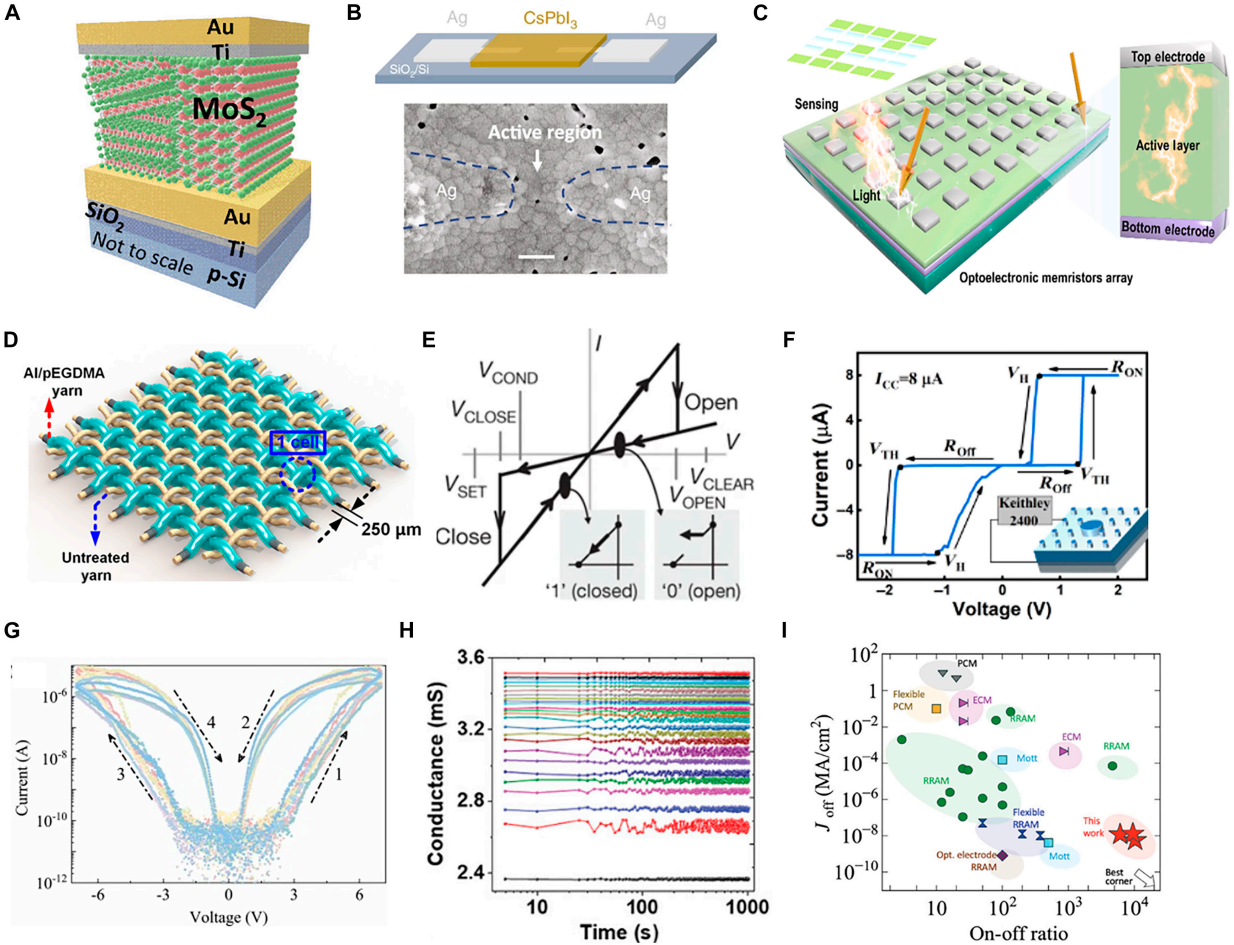
(A) Device schematic of Ti/Au/MoS_2_/Au memristor [46]. Copyright 2022, ACS. (B) Schematic and scanning electron microscopy image of Ag/CsPbI3/Ag memristor [49]. Copyright 2020, Zhu et al. (C) Schematic of the optoelectronic memristors array [53]. Copyright 2021, Elsevier Ltd. (D) Illustrative representation of the fabricated memory device utilizing cross-linked Al/pEGDMA-coated yarns [55]. Copyright 2017, ACS. (E) Idealized memristor-based electrical characteristics, with distinct voltage thresholds that switch between the low-resistance (logical “1”) and high-resistance (logical “0”) states [67]. Copyright 2010, Macmillan Publishers Limited. (F) Representative *I*–*V* characteristics of ANO threshold devices under an 8-μA compliance current, with the test circuit [68]. Copyright 2024, Zhao et al., InfoMat published by UESTC and John Wiley & Sons Australia Ltd. (G) Switching behaviors of multiple S-InSe memristors [69]. Copyright 2024, Elsevier Ltd. (H) Stable retention of conductance states during potentiation [52]. Copyright 2024, Park et al. (I) Current density in off-state versus switching ratios [70]. Copyright 2024, Wiley-VCH GmbH.

In memristors based on 2-dimensional materials, resistance switching typically arises from the formation of conductive filaments within the active layer [44,45]. A representative device structure is the Ti/Au/MoS_2_/Au configuration, where MoS₂ thin films synthesized by chemical vapor deposition (CVD) serve as the functional layer, and Ti/Au layers are used as the top electrodes (Fig. 2A) [46]. The use of MoS₂, a layered transition metal dichalcogenide (TMD), plays a critical role in the device’s memristor-based behavior. Its intrinsic layered structure allows for efficient ion migration and charge trapping and detrapping at defect sites, which facilitates stable and repeatable resistance switching. Meanwhile, the Au bottom electrode provides excellent chemical stability and low contact resistance, while the Ti interlayer enhances adhesion and modulates the Schottky barrier at the MoS₂ interface, further improving switching uniformity and reducing cycle-to-cycle variability. Owing to these material synergies, the device exhibits reliable memristor-based performance. In addition to conductive filament mechanisms, resistance switching can also arise from the redistribution of intrinsic ion vacancies within the switching layer [47,48]. A device using an Ag/CsPbI_3_/Ag structure (Fig. 2B) demonstrates volatile switching behavior caused by the migration of iodine ions under an electric field [49]. The device exhibits volatile resistance switching behavior, primarily governed by the migration of iodine ions (I^−^) within the CsPbI_3_ lattice under an applied electric field. Due to the relatively low activation energy for halide ion migration in perovskite structures, iodine ions can drift through the lattice and accumulate near the electrode interfaces, modulating the local electrostatic potential and conductivity. When the field is removed, the ions gradually diffuse back, leading to the volatile (nonretentive) nature of the switching. This ion migration is also facilitated by the presence of intrinsic halide vacancies and the soft ionic lattice of CsPbI_3_, which allows for fast, field-driven ionic motion. Such behavior enables fast switching speed and reversibility.

The inputs to logic gates are not limited to electrical signals; various external stimuli can also serve as control inputs [50,51]. In optoelectronic logic gates, achieving light-controlled resistance switching requires appropriate material selection and interface design [52]. Devices employing ZnO as the active layer and Ag as the top electrode, configured in an ITO (indium tin oxide)/ZnO/Ag structure (Fig. 2C), demonstrate this principle effectively [53]. Illumination generates electron–hole pairs within the ZnO layer, modulating its conductivity and enabling reconfigurable logic functions. This optoelectronic synergistic effect enabled the memristor array to perform reconfigurable logic operations with enhanced flexibility. Extending memristor-based logic into mechanically compliant form factors, recent work on textile-integrated devices shows that resistance switching can be embedded in wearable, low-power platforms [54]. A textile-form memristor (ETM) was fabricated in which cotton yarn bundles coated with aluminum act as woven electrodes, and an initiated chemical vapor deposition (iCVD) poly(ethylene glycol) dimethacrylate (pEGDMA) thin film serves as the switching layer (Fig. 2D) [55]. The Al/pEGDMA-coated yarns were interwoven with untreated cotton yarns to form a cross-array. The ETM exhibited excellent switching characteristics, including low operating voltage, high data retention, and durability. This structure achieves robust resistance switching behavior due to the formation and rupture of carbon filaments within the pEGDMA layer, which maintains high stability even under mechanical stresses such as bending and washing. These features make textile memristors promising candidates for implementing logic gates in flexible and wearable computing systems.

The performance and functionality of memristor-based logic gates are fundamentally determined by the interplay between material properties and device architecture. The selection of functional materials—ranging from layered 2-dimensional compounds and halide perovskites to photosensitive and polymer-based films—directly governs key switching characteristics such as speed, retention, volatility, and energy efficiency. Meanwhile, structural designs, including MIM configurations, crossbar arrays, and flexible fiber-based geometries, enable enhanced field modulation, scalability, and mechanical robustness. These material–structure synergies not only improve the stability and efficiency of resistance switching but also broaden the input modalities and application scenarios, paving the way for adaptive, low-power, and reconfigurable logic systems in future electronic platforms.

### Memristor performance

In addition to materials and structure, the performance metrics of memristors are also an important foundation for the implementation of logic gates [56,57]. The distinction between HRS and LRS (the switching ratio) determines the accuracy of logic signals [58–60]. The current–voltage characteristics reflect the device’s nonlinear response capability, while retention time and cycling stability are key to the long-term reliable operation of logic gates. In practical logic operations, memristors must maintain consistency of states after multiple resistance switches to ensure the accuracy of the logic results [61,62]. At the same time, the environmental adaptability of the device, such as its response to humidity and temperature, also determines its feasibility in practical applications [63,64].

The resistance switching behavior of memristors enables direct implementation of logic functions at the device level [65,66]. One representative demonstration involves logic computation based on material implication (IMP), where the device’s resistive state directly participates in the logic process. This approach—often referred to as “stateful logic”—enables data storage and logic execution to occur within the same physical element, as illustrated in Fig. 2E, offering a compact and energy-efficient alternative to traditional logic architectures [67]. This memristor is composed of a titanium oxide (TiO_2_) functional layer sandwiched between Pt electrodes, forming a nanoscale cross-array structure. It exhibits stable bipolar resistance switching characteristics, with the ability to switch between HRS and LRS, having a clear voltage threshold. The stable, threshold-dependent resistive switching behavior of memristors provides precise control over logic states, which is essential for implementing reliable in-memory logic operations. A device employing an antiferroelectric AgNbO_3_ (ANO) thin film demonstrates consistent switching from an HRS to an LRS under a current compliance of 8 μA (Fig. 2F), confirming its suitability for logic-level control and integration [68]. Building on this, another memristor design utilizes a suspended 2-dimensional S-InSe structure, offering further improvements in switching uniformity and device reliability. This configuration exhibits high ON/OFF ratios, forming-free behavior, and low variability across switching cycles (Fig. 2G) [69]. These memristors exhibit a high ON/OFF ratio (10^5^), forming-free operation, high yield (97%), and low cycle-to-cycle variability (7.4%), which can enhance the stability and reliability of memristor-based logic gates. Multilevel conductance states are crucial for memristor-based logic gates as they enable multiple logical states within a single device, enhancing the information storage capacity and computational efficiency, thereby improving the complexity of logic operations and neuromorphic computing. While switching uniformity is essential for reliable logic operations, the ability to retain multiple conductance states over time is equally important for enabling nonvolatile and high-density logic computation. Figure 2H illustrates the nonvolatile retention properties of multilevel conductance states in vapor-transport-deposited (VTD) Sb_2_S_3_ memristors [52]. This device exhibits stable and distinct multilevel conductance states across multiple testing cycles, demonstrating reliable and controllable multilevel resistive switching under various compliance currents. Expanding the capability of logic circuits, multilevel resistance states with strong retention characteristics allow multiple logic levels to be encoded in a single memristor, significantly improving integration density and circuit versatility. One demonstration involves a HfO_2_-based memristor incorporating magnetron-sputtered Ti_2_AlN MAX phase layers, which exhibits reliable switching behavior and well-defined conductance states (Fig. 2I) [70]. The device exhibits an ultralow reset current density (<10^−8^ MΩ·cm^2^) and a high ON/OFF ratio (6,000). For memristor-based logic gates, a lower reset current significantly reduces energy consumption during frequent switching operations. This improvement in energy efficiency and thermal management enables a greater number of memristor units to be integrated within a given chip area. As a result, it not only supports the miniaturization of computing hardware but also enhances the scalability and parallelism of memristor-based logic and memory architectures. Such integration is particularly advantageous in applications requiring high-density, low-power logic operations, paving the way for future intelligent systems that combine compact size with computational efficiency.

Memristors based on different material systems exhibit distinct advantages and challenges in terms of logic reliability, switching speed, scalability, and power consumption. For instance, Cu_2_S-based 2-dimensional memristors leverage the rapid intrinsic migration of Cu^+^ ions to achieve ultralow power consumption (as low as 1 μW at 100 mV) without requiring external ion insertion while also demonstrating excellent switching speed and cycling stability [71]. However, their long-term durability and large-scale integration still face limitations. Textile memristors, constructed from reconfigurable Ag/MoS₂/HfAlO*_x_*/carbon nanotube architectures, integrate both nonvolatile synaptic plasticity and volatile neuron-like behavior [29]. Their energy consumption per spike can be as low as 1.9 fJ, offering remarkable efficiency and structural flexibility, with promising potential for use in large-scale logic gate implementations. Optoelectronic memristors, incorporating perovskite switching layers and TiO_2_ interfacial films via atomic layer deposition, achieve low V_SET_ (+0.24 V) and low power operation (~0.7 μW) with stable nonvolatile switching behavior. These devices also support multimodal logic operations under combined optical and electrical stimuli, showing balanced performance in logic reliability, power efficiency, and integration capability [72]. In summary, 2-dimensional memristors excel in speed and energy efficiency, textile memristors are advantageous for ultralow-power and flexible designs with high potential in scalable logic, while optoelectronic memristors offer strong stability and support for complex, multimodal logic processing.

The performance of these studies’ materials and devices highlights the representative mechanisms and design strategies underlying memristor-based logic gates. Priority was given to studies involving a variety of material systems, including 2-dimensional materials, perovskites, polymers, and optoelectronic functional materials, which demonstrate both the resistive switching mechanisms and durability of memristors. The presented device architectures are diverse, encompassing vertical stacks, planar configurations, crossbar arrays, and fiber-based fabrics, reflecting current research trends aimed at enhancing switching performance while integrating logic reconfigurability with mechanical flexibility.

## Basic Boolean Logic Gates

In traditional digital circuit design, logic gates are typically based on CMOS technology, realized through the combination of transistor circuit elements [73,74]. However, with the rapid growth of modern computing demands, particularly in fields such as artificial intelligence (AI) and the Internet of Things, there are higher requirements for computing power, efficiency, and integration density [75–77]. Traditional CMOS technology faces challenges such as high-power consumption, limited area, and complex manufacturing processes [78,79]. As a result, memristor-based logic gates have gradually become a hot research topic [80,81].

Due to their nonvolatility, multi-level resistance tunability, and state retention characteristics, memristors are considered key devices for achieving the integration of logic computation and information storage [78,82,83]. Unlike traditional CMOS devices, which only exhibit transient on/off states, memristors can present stable and programmable resistance states through physical mechanisms such as ion migration and oxygen vacancy redistribution, and are able to retain these states even when powered off [84]. In the design of logic gates, memristors can be configured into simple resistive network structures. By exploiting voltage division effects under different resistance states and combining with threshold-based voltage comparator circuits, basic Boolean logic functions such as AND, OR, and NOT can be realized [85,86]. Furthermore, memristors support the implementation of more complex logic units, such as those based on material IMP logic. As a functionally complete logic, IMP can be used to construct arbitrary Boolean functions, providing a theoretical foundation for memristor-based logic computation [87,88].

**Fig. 3.**
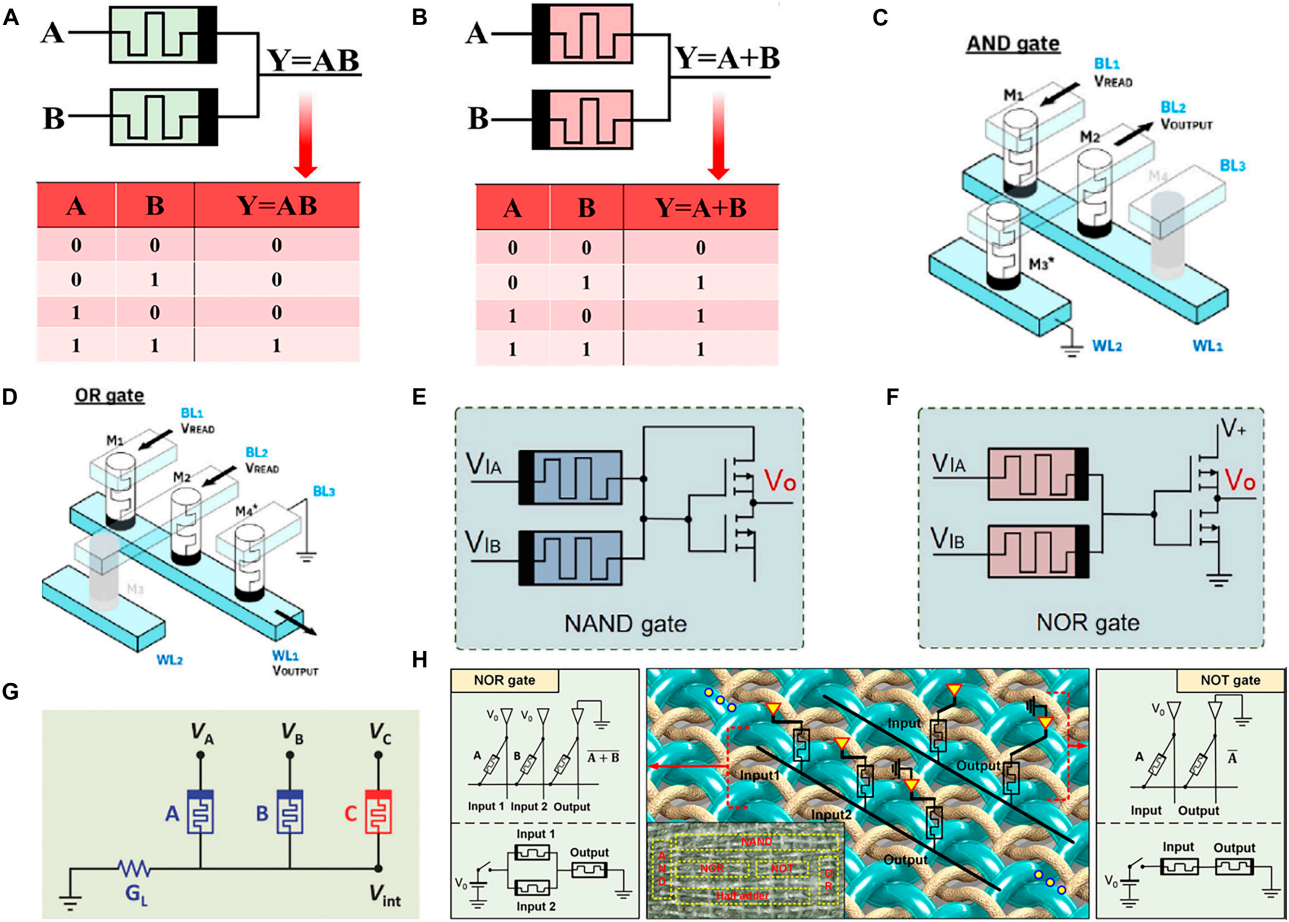
(A) AND logic gate along with its corresponding truth table. (B) OR logic gate along with its corresponding truth table [65]. Copyright 2024, ACS. (C) AND gates constructed using specific elements in a crossbar structure. (D) OR gates constructed using specific elements in a crossbar structure [89]. Copyright 2022, ACS. (E) NAND logic gate implemented using memristors. (F) NOR logic gate implemented using memristors [91]. Copyright 2024, Elsevier Ltd. (G) RRAM (resistive random access memory) circuit featuring 2 input devices (A and B), one output device (C), and a load resistor with conductance G_L_ [95]. Copyright 2018, WILEY-VCH Verlag GmbH & Co. KGaA, Weinheim. (H) Schematic of NOR and NOT logic-in-memory operations and their equivalent circuits within the crossbar array using the MAGIC architecture. The inset provides a conceptual image of how each logic gate is integrated into the fabric [55]. Copyright 2017, ACS.

One practical approach to implementing memristor-based logic gates is through voltage division across devices with different resistance states. This method simplifies the circuit design by directly mapping input logic states to output voltages. Based on this principle, a device based on a graphene-doped polyvinyl alcohol (PVA) composite and structured as Ag/PVA-Gr/Ti has been demonstrated to implement an AND logic gate (Fig. 3A) [65]. The AND logic gate is composed of 2 memristors with opposite polarities. When both inputs (A and B) are logic “1”, both devices switch to the LRS, producing a high output. If either input is logic “0”, at least one memristor remains in the HRS, resulting in a low output. Similarly, the OR logic gate uses 2 oppositely polarized memristors, as shown in Fig. 3B. A high output is generated when either input is logic “1”, while both remaining in HRS yields a low output. In addition to voltage division-based logic gates, material IMP operations offer another effective strategy. This approach exploits the intrinsic state-dependent switching of memristors to realize logic functions. A typical implementation involves configuring 3 memristors (M1, M2, and M3) in a crossbar array to construct an AND gate (Fig. 3C) [89]. The resistance states of M1 and M2 represent logic inputs “0” and “1”, while M3 is initialized to an intermediate state to regulate voltage distribution. When both M1 and M2 are in LRS, voltage drops mainly across M3, producing a high output. If either is in HRS, the output remains low. Experimental results verified accurate logic performance. Figure 3D illustrates the OR gate implementation, where M1 and M2 serve as inputs and M4 serves as the intermediate device. A high output is generated if either M1 or M2 is in LRS; otherwise, the output remains low. Compared to IMP logic, the voltage division-based ratio logic offers a more intuitive and hardware-efficient implementation by directly mapping input voltages to output states. However, IMP logic allows for more complex, reconfigurable logic operations and better scalability in crossbar arrays, as it decouples logic execution from physical wiring. Thus, ratio logic is suitable for simple, low-power logic gates, while IMP logic provides a foundation for more programmable, in-memory computing architectures.

Traditional NAND logic gates are typically implemented using CMOS technology. However, with the increasing complexity of integrated circuits and the demand for low power consumption and high density, memristor-based NAND gates have gained significant attention due to their simple structure, low power consumption, and the integration of both logic and memory functions [90]. Integrating memristors with CMOS technology can further enhance circuit performance. As shown in Fig. 3E, Lin et al. [91] implemented a NAND logic gate by combining a memristor-based AND gate with a CMOS NOT gate. The memristor encodes logic inputs through its resistance state, and the CMOS inverter provides output inversion. Using the same method, Fig. 3F illustrates a NOR logic gate formed by connecting a CMOS NOT gate to a memristor-based OR gate. In both cases, the hybrid structure enables efficient realization of logic functions by leveraging the advantages of memristors and CMOS devices. Compared to pure memristor logic gates, hybrid CMOS–memristor circuits offer enhanced signal integrity, faster switching speed, and better compatibility with existing CMOS infrastructure. However, they may increase design complexity and occupy more chip area due to the additional CMOS components. In contrast, pure memristor logic gates provide ultrahigh density and low power consumption, making them more suitable for in-memory computing and large-scale parallelism, but they often suffer from signal degradation, slower switching, and limited logic flexibility without auxiliary circuitry.

The design mentioned above fully utilizes the resistance switching characteristics of memristors, combined with the high gain characteristics of CMOS devices, to improve the quality of the output logic signals [92,93]. However, such designs still rely on traditional CMOS processes, which pose certain limitations in terms of circuit area and power consumption [94]. To further optimize logic gate implementation, Fig. 3G shows a stateful logic architecture entirely based on memristors, eliminating the need for CMOS components [95]. It demonstrates a NAND gate composed of 3 memristors (A, B, and C) and a load resistor. The resistance states of A and B represent inputs, while C serves as the output. When both inputs are logic “1”, the internal voltage remains below the switching threshold, keeping C in the HRS. In all other cases, C switches to low resistance, thus realizing NAND logic through resistance-state interactions and voltage control. In addition to stateful logic, another type of pure memristor-based logic, known as the MAGIC (memristor-aided logic) scheme, has been developed (Fig. 3H) [55]. In this approach, the NOR gate is implemented using a memristor crossbar, where inputs M1 and M2 are in parallel and the output M3 is in series. Only when both inputs are at HRS does M3 receive sufficient voltage to switch to LRS; otherwise, the output remains in HRS. These implementations underscore the potential of fully memristor-based architectures to realize a wide range of logic functions solely through resistance-state manipulation and voltage control. By eliminating the need for CMOS transistors, such designs significantly reduce circuit complexity and power consumption, paving the way for highly compact, energy-efficient, and scalable in-memory computing systems.

Various memristor-based logic gate implementation approaches exhibit distinct design strategies and system-level characteristics, highlighting the multi-level application potential of memristors, ranging from basic logic construction to the integration of logic and memory. Simple voltage-divider-based designs for AND and OR gates rely on the direct switching behavior of memristors. These structures are relatively straightforward and suitable for verifying the feasibility of basic logic functionality using memristors; however, they offer limited integration density and scalability. In contrast, logic gates embedded within crossbar architectures introduce parallel computing capabilities at the array level. These designs utilize the conductance states of memristors to represent Boolean variables and perform logic operations under shared line control, enabling higher parallelism and hardware scalability. They are well-suited for constructing dense logic matrices. The MAGIC architecture represents the most integrated approach. It not only realizes logic operations but also tightly couples them with the memristor array itself. Compared with traditional gate-level logic, this architecture significantly reduces data movement latency and improves energy efficiency, making it particularly promising for edge computing and neuromorphic hardware applications.

## Optoelectronic Logic Gates

With the rapid development of computer technology, traditional electronic logic gates have gradually shown limitations in processing multi-modal data due to constraints in power consumption and processing speed [96,97]. As a new type of functional device, memristors, with their unique resistance switching characteristics and nonvolatility, show great potential in combining logic operations and memory functions. However, relying solely on electrical inputs is no longer sufficient to meet the current demands [3,98,99]. In recent years, optoelectronic input memristor logic gates have gradually become a research hotspot. By directly using optical signals as inputs for logic operations, they not only significantly enhance the response speed of logic gates but also expand the application scope of memristors in the field of optoelectronic computing [100,101].

**Fig. 4.**
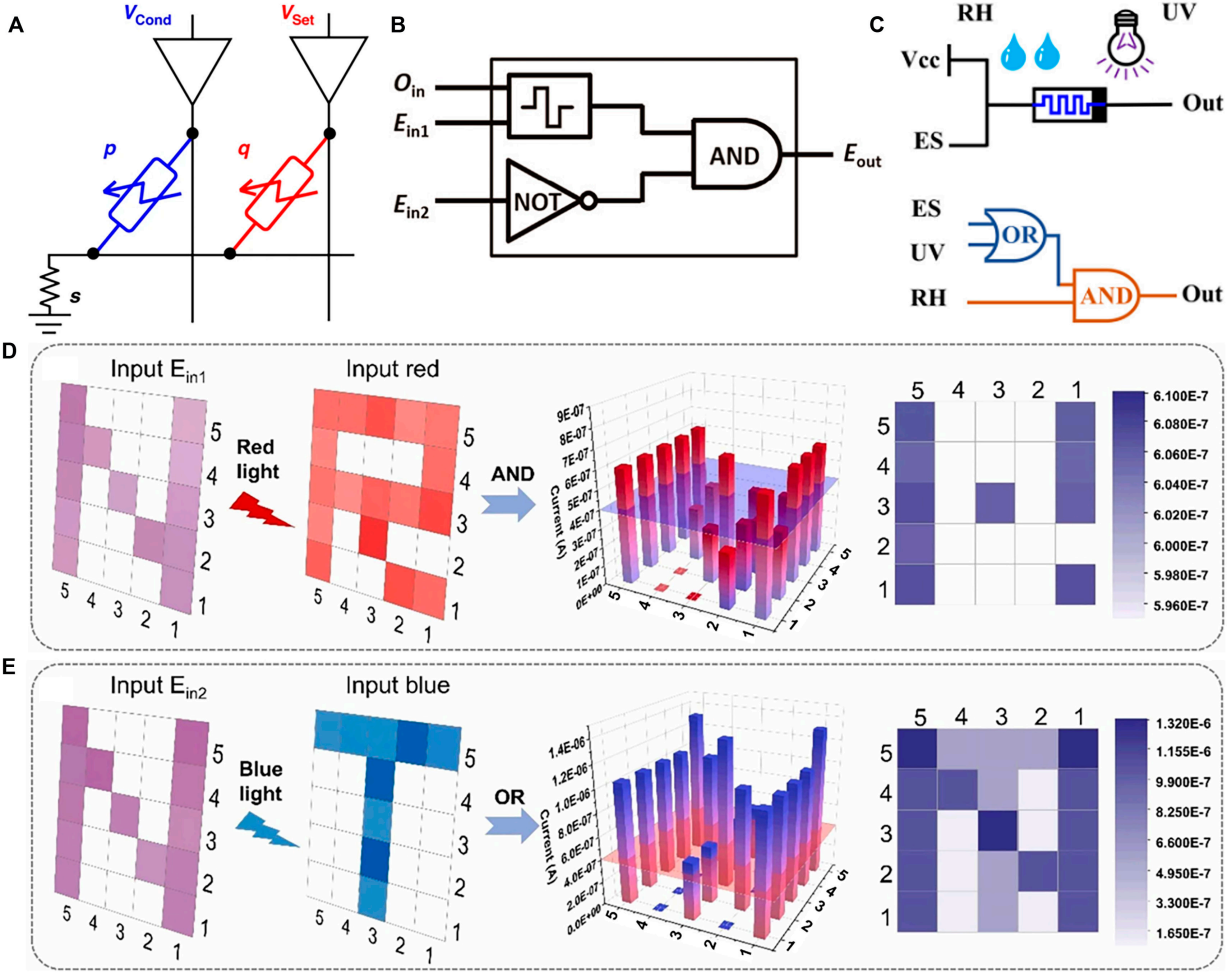
(A) Illustration of an in-memory IMP operation using a set of photo-memristors triggered by light stimuli [84]. Copyright 2023, Fu et al. (B) Complicated memristor-based logic gate incorporating the “NOT” operation, which reconfigures the memristor-based logic gate into a blank device. Ein2 is the second electrical input used to reformat the device [106]. Copyright 2017, ACS. (C) Three-person voting device logic gate circuit diagram featuring a one-vote veto logic function [109]. Copyright 2024, ACS. (D) Logic output of the “AND” operation resulting from the integration of the letters “N” and “R”. (E) Logic output of the “OR” operation resulting from the integration of the letters “N” and “T” [112]. Copyright 2022, Elsevier Ltd.

Optical input logic gates leverage the sensitivity of special materials in memristors to light stimuli. By utilizing light-induced carrier separation or photo-induced resistance changes, these gates perform logic operations [102,103]. Compared to traditional electronic input logic gates, optoelectronic input logic gates offer faster response times and lower power consumption. Additionally, by combining multi-modal inputs of both optical and electrical signals, the flexibility and functionality of memristor logic gates can be further enhanced [104,105]. One implementation involves applying optoelectronic stimuli to material IMP logic gates. A device utilizing a graphene/oxygen-doped MoS₂/graphene heterostructure demonstrates both logic gate functionality and integrated memory computing capability (Fig. 4A) [84]. This photo-memristor utilizes a combination of light and voltage stimuli to achieve nonvolatile response switching and logic operations. They designed an IMP logic gate, where both input and output are encoded through the light response states of the photo-memristor. The IMP logic gate operation takes advantage of the multi-state light response characteristics of the memristor. In the IMP operation, 3 photo-memristors (denoted as p, q, and s) are connected in an array to form the logic circuit. The photo-memristor s is initialized to HPS (a high photoresponse state), while p and q switch their response states based on the input logic through a combination of light and voltage control. Specifically, when q is in the LPS (a low photoresponse state), the applied combination of voltage and light can force its state to switch to HPS. However, when q is in HPS, the voltage is insufficient to trigger a state change, ensuring that its state remains unchanged, thereby performing the IMP logic operation.

Building on these light-responsive mechanisms, optoelectronic input memristors have evolved beyond basic Boolean logic. Their ability to respond to both optical and electrical stimuli significantly enhances the reconfigurability and versatility of logic gate design. Memristors have been developed to function as optical logic units, capable of not only performing conventional Boolean operations but also supporting advanced functions with hybrid optical–electrical inputs. A photo-memristor incorporating an ITO/CeO_2−*x*_/AlO*_y_*/Al multilayer architecture demonstrates multifunctional and reconfigurable logic behavior, allowing dynamic switching between AND, OR, and NOT logic depending on the applied stimuli. This photo-memristor combines electrical and optical signal inputs, achieving resistance-state switching through the migration and capture of oxygen vacancies at the interface. Figure 4B demonstrates that after introducing a second electrical signal input, Ein2, the memristor can perform NOT logic operations through an electro-optic hybrid mode [106]. In this operation, when the Ein2 signal is applied, the output current is forced to be suppressed to the LRS, regardless of the previous logical state of the logic unit. The final output signal is determined by the 3 input signals. This implementation illustrates that by leveraging electro-optic co-modulation, the logic state of the memristor can be actively and reversibly reset, enabling more flexible and reconfigurable logic behavior. Such dynamic control not only enhances the adaptability of logic circuits to complex input patterns but also lays the groundwork for developing multifunctional, low-power logic units suitable for integration into advanced computing systems.

The combination of optical and electrical signals offers a flexible input modality for memristor logic gates and enhances the diversity of logic operations. However, in complex environments, such dual-input schemes may be insufficient for intelligent decision-making tasks [107,108]. To overcome this limitation, researchers have introduced environmental parameters—such as humidity—into logic computation frameworks. By integrating optical, electrical, and humidity signals, memristor-based circuits can achieve complex multimodal logic operations, where humidity serves as a veto input to control the output state (Fig. 4C) [109]. The MXene component allows the device’s performance to be influenced by humidity, enabling the combination of optical, electrical, and humidity signals. By using humidity as the core of a “veto” mechanism, this design achieves higher-level logic operations. By representing the relative humidity (RH), electrical signal, and optical signal as In1, In2, and In3, respectively, and setting the reading voltage to 0.1 V, the output threshold of the logic circuit is defined as 3.5 μA. Specifically, when the RH value exceeds 70% (In1 = 1), the output remains 0, regardless of whether the electrical or optical signal states meet the logical requirements, demonstrating the veto power of RH. When RH is below 70%, and at least one of the electrical or optical signals is in the “1” state, the logic circuit outputs “1”. This multimodal logic design significantly improves the adaptability of memristor circuits to complex environments by integrating electrical, optical, and humidity signals as logic inputs. It enables conditional decision-making and environment-aware computation, laying the foundation for applications in intelligent sensors, edge computing, and low-power wearable electronics.

To further assess the practicality of optoelectronic inputs and their suitability for low-power applications, researchers have introduced logic architectures responsive to different wavelengths of light [110,111]. One such configuration employs an Ag/Ta_2_O_5−*x*_/Ta_2_O_5_/N-Si layered structure, enabling logic and in-memory computing operations under combined optical and electrical stimuli (Fig. 4D) [112]. Figure 4D and E shows the implementation processes of logic “AND” and logic “OR” operations, respectively. In the logic “AND” operation, both the red-light signal and the electrical signal Ein1 serve as inputs. The electrical signal Ein1 selects the letter “N”, and the red-light signal selects the letter “R” as array inputs. Only when both signals are applied does the device’s conductivity exceed the preset threshold (Vth = 400 nA), resulting in a logic output of “1”; otherwise, the output is “0”. The total energy consumption is as low as 4.5 nJ, indicating its suitability for low-power integrated memory computing applications. In the logic “OR” operation, the blue light selects the letter “T”, and Ein2 selects the letter “N” as array inputs. As long as either input is active, the device reaches the conductive threshold and outputs logic “1”. These implementations not only demonstrate the feasibility of executing reliable Boolean logic within optoelectronic arrays but also highlight the potential of such devices to serve as multifunctional logic units capable of low-energy, in situ data processing under multimodal stimuli—laying the foundation for future heterogeneous, high-efficiency computing systems.

Optoelectronic memristor logic gates, driven by light stimuli, represent a promising emerging architecture that differs fundamentally from conventional voltage-driven memristor logic gates in both structural design and operational mechanism [113,114]. Traditional memristor logic gates rely solely on electrical voltage pulses for programming and reading, which limits their scalability due to complex wiring and a finite number of input channels [72,114]. In contrast, optoelectronic memristors introduce light as a noncontact, tunable input [45,52]. By using mechanisms such as photogenerated carrier excitation or light-induced ion migration, they achieve controlled switching between resistance states [115]. This enables wavelength-selective, spatially resolved, and parallel logic operations, overcoming the interconnection bottlenecks of purely electrical systems [116,117]. The synergistic use of optical and electrical inputs also enhances reconfigurability, allowing the dynamic adjustment of logic functions without changing the physical device structure [118,119]. These characteristics make optoelectronic memristors highly suitable for applications requiring low power consumption, high parallelism, and flexibility, such as wearable electronics, neuromorphic vision, and edge intelligence [87]. However, their strong sensitivity to environmental variables, particularly ambient light, may introduce significant challenges in practical deployment. Uncontrolled or unintended illumination can trigger undesired resistance switching, leading to false logic outputs or data corruption. Variations in ambient lighting conditions—such as fluctuations in indoor or outdoor light intensity, temperature-dependent responsivity shifts, or spectral overlap with the operational wavelengths—can compromise the consistency and repeatability of logic operations. Despite current limitations, the unique light-controlled programmability and multidimensional input capacity of optoelectronic memristor logic gates offer a novel and powerful pathway toward next-generation heterogeneous and reconfigurable computing architectures.

## Combinational Logic Circuits

Basic logic gates are the cornerstone of digital circuits, providing the essential modules for constructing complex logic operations. In practical applications, many complex computational tasks often require the clever combination of multiple logic gates to achieve the desired outcome. Memristors, with their unique deep integration of logic and memory functions, show great potential and a promising future in the field of combinational logic implementation [107,120]. Compared to traditional CMOS logic circuits, memristor-based combinational logic gates not only effectively reduce hardware complexity but also enable more efficient and precise computation processes by leveraging stateful logic [121,122].

### Construction of basic combinational logic

**Fig. 5.**
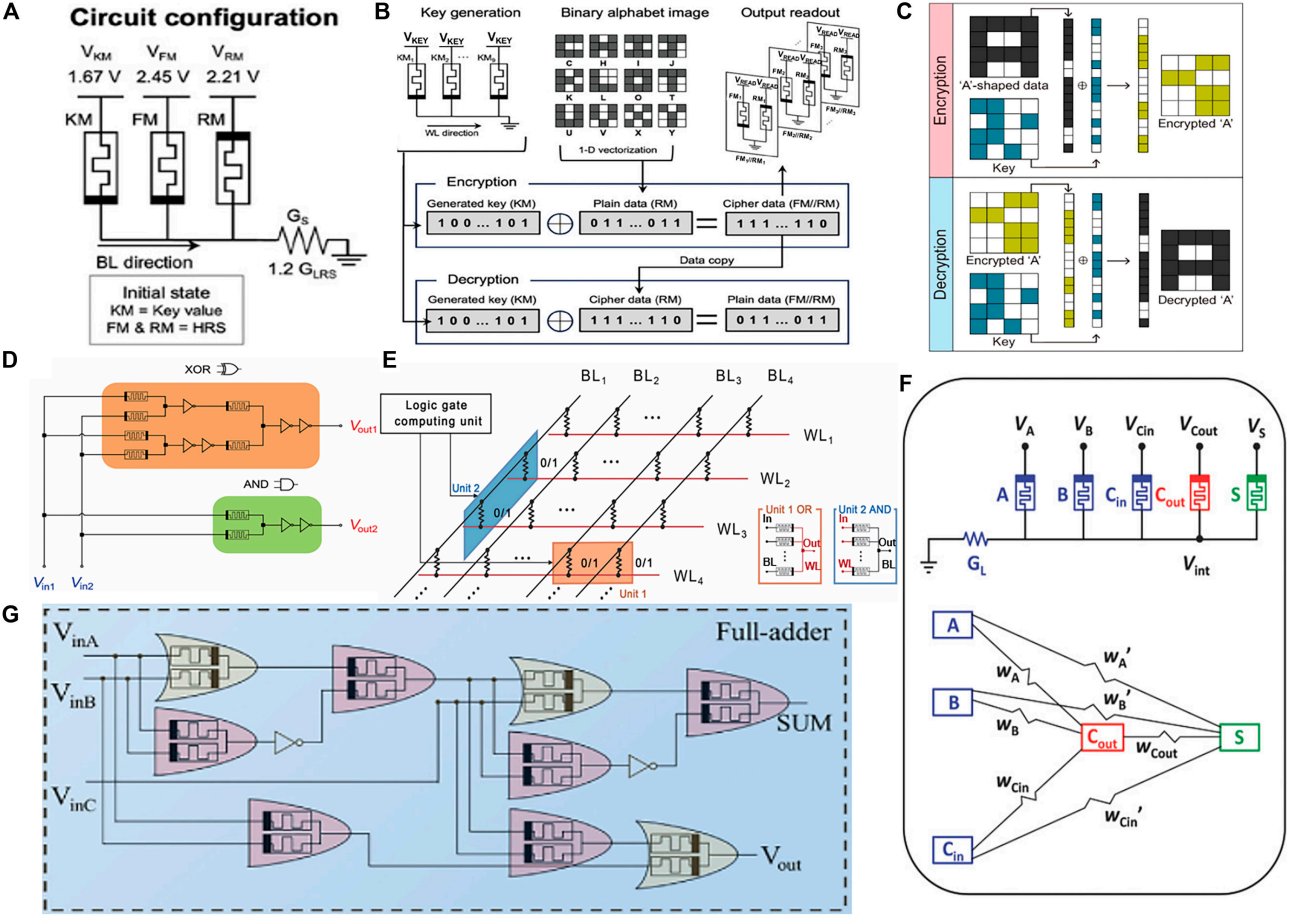
(A) Schematic diagram of the circuit configuration and voltage application for the XOR gate. (B) After 1D vectorization, 3 × 3 binary alphabet images can be encrypted and decrypted using DP-CBA (dual-polarity crossbar array), with a 1 × 9 vector key applied for the encryption and decryption process [123]. Copyright 2024, Wiley-VCH GmbH. (C) The encryption and decryption of A-shaped data are carried out using a key equivalent to PUF 1 (physically unclonable function), with the process being executed via an XOR operation based on the dc logic scheme [125]. Copyright 2024, Woo et al. (D) Structure of the designed half-adder implemented using al-atomristor logic gates and an inverter. (E) All-atomristor logic gates in the crossbar memristor array, with the insets depicting the circuits of the parallel logic gates [132]. Copyright 2022, Tsinghua University Press. (F) RRAM circuit and its corresponding equivalent 2-layer perceptron network for the 1-bit full adder (FA) [95]. Copyright 2018, WILEY-VCH Verlag GmbH & Co. KGaA, Weinheim. (G) Memristor-based FA circuit [133]. Copyright 2022, Elsevier Ltd.

Extending beyond basic logic operations, efforts have been directed toward developing more advanced logic functions using memristor-based devices. A representative approach involves a crossbar array configured with bipolar Ta/HfO_2_/RuO_2_ (THR) memristors, designed to efficiently execute XOR logic operations and support integration into memory encryption systems (Fig. 5A) [123]. The design uses anti-parallel forward and reverse memristors (FM and RM), with input logic encoded by the resistance states of a key memristor (KM) and RM. The XOR result is stored in FM after one-step conditional switching. Figure 5B shows the encryption process: A binarized image is vectorized and encrypted via bitwise XOR with a 9-bit random key from the KM, and decrypted by repeating the operation, restoring the original data. This showcases the potential of memristor logic in secure and efficient data processing. In practical applications, to further enhance the flexibility and energy efficiency of the encryption logic, researchers have begun exploring the performance optimization of different memristor materials and the design of novel logic architectures [124]. An alternative strategy focuses on utilizing a Cu_0.3_Te_0.7_/HfO_2_(CuTeHO) memristor crossbar array, which supports low-power logic operations while maintaining data confidentiality (Fig. 5C). This architecture exemplifies ongoing efforts to improve energy efficiency and functional reliability in memristor-based encryption circuits [125]. The CuTeHO memristor utilizes its unique dual-voltage-condition logic method, combined with randomly generated keys, to successfully perform the encryption and decryption of the letter “A” data. The encryption operation is also implemented through the XOR logic of memristors. They first “map” the input data to the resistance states of the memristor array and perform bitwise XOR operations with the generated key to produce the encrypted data. Then, decryption is performed using the same key, successfully restoring the original input data. This encryption logic scheme validates the effectiveness of the CuTeHO memristor system, particularly in terms of data confidentiality and energy efficiency, providing a potential solution for high-density memory computing integration applications and hardware security systems.

Building upon the promising application of memristors in basic and security-oriented logic operations, researchers have also explored their potential in constructing more complex combinational logic circuits [126,127]. Among these, half-adders and full-adders (FAs) are fundamental components for arithmetic computation. Traditional CMOS implementations of such units require multiple interconnected gates and numerous transistors, which increases hardware complexity and power demands [128,129]. Memristor-based circuits, which integrate both logic and memory capabilities within a single device, offer a streamlined solution with reduced area and power demands compared to traditional CMOS-based designs [130,131]. The bistable resistance of monolayer MoS₂ memristors lends itself well to arithmetic operations. A half-adder based on this material successfully performs binary addition using XOR and AND logic functions (Fig. 5D) [132]. This design successfully implements binary addition by utilizing the bistable resistance characteristics of the memristor, combining XOR and AND logic operations. In this setup, the Vout1 signal corresponds to the output of the XOR operation, while the V_out2_ signal corresponds to the output of the AND gate operation. When both input signals, V_in1_ and V_in2_, are high (logic “1”), both V_out1_ and V_out2_ outputs are high (logic “1”), indicating a carry. In other cases, V_out1_ and V_out2_ are low (logic “0”). They also designed a logic unit based on a memristor array (Fig. 5E) to implement complex combinational logic operations. This study successfully achieved parallel logic gate operations by using a 2-dimensional monolayer MoS₂ memristor as the core device, combined with an array design. The resistance state of the memristors is controlled by adjusting the voltage, allowing for programming of multiple memristor units in the array. This approach enables the construction of complex logic circuits, such as adders and XOR gates. Building upon the structure of half-adders, FA circuits can be realized using memristor-based logic gates to perform more complex arithmetic tasks. As shown in Fig. 5G, the circuit executes binary addition by integrating XOR and AND logic functionalities within the same architecture. This circuit uses V_inA_, V_inB_, and V_inC_ as input signals, with an additional carry input compared to the half-adder. The output signals are SUM and V_out_ [133].

As the complexity of computation increases, implementing the FA function through combinations of basic logic gates may face limitations in terms of efficiency and integration density [134,135]. To reduce the number of required devices and enhance logic efficiency, some designs combine memristors with state-based networks [136]. One approach utilizes linearly separable majority functions together with linearly inseparable parity functions to compute the SUM and CARRY logic of an FA. This configuration is illustrated in Fig. 5F, where the structure effectively performs single-bit addition within a compact architecture [95]. The circuit consists of 3 inputs (V_A_, V_B_, and V_Cin_) and 2 outputs (V_Cout_ and VS), where V_Cout_ represents the majority logic of the inputs and VS represents the parity logic. By adjusting the synaptic weights of the memristors and the applied voltage, this design computes V_Cout_ in the first step and VS in the second step. The entire FA logic is completed in just 2 steps. This architecture reduces the area and power consumption of the combinational logic circuit, further highlighting the advantages of using memristors in logic circuit design.

Combinational logic circuits based on memristors leverage their nonvolatility and tunable resistance characteristics to implement more complex logic functions such as XOR gates, half-adders, and FAs through carefully designed circuit structures. Compared to traditional approaches, these designs eliminate the need for complex transistor stacks by using the resistance states of memristors as logic variables, significantly reducing device count and integration area. Moreover, certain logic architectures incorporate crossbar arrays and weight mapping schemes, enabling reconfigurability and parallelism in logic operations, thus highlighting the advantages of memristors in building compact and efficient logic units.

### Array level logic

**Fig. 6.**
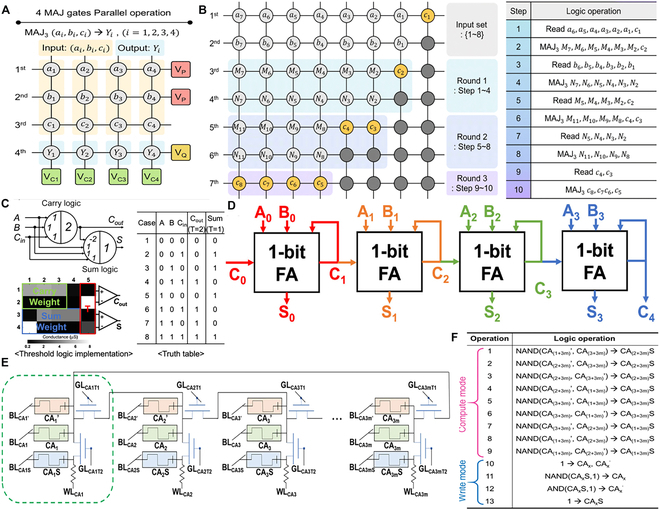
(A) Block diagram illustrating the parallel operation of 4 MAJ gates. (B) Block diagram and logical operation procedures for the 8-bit KSA (Kogge–Stone adder) [141]. Copyright 2025, Royal Society of Chemistry. (C) Threshold logic model and its implementation of a 1-bit FA in a memristor crossbar array. (D) Implementation of a 4-bit RCA using a series of 1-bit FAs [144]. Copyright 2024, ACS. (E) Implementation of a 4-bit RCA using a series of 1-bit FAs. (F) Circuit of the 1D CA, with the subcircuits enclosed in a green box representing a single basic cell [147]. Copyright 2023, Liu et al.

With the continuous development of memristors in the field of logic computation, researchers are actively exploring more direct and efficient implementation approaches that utilize array-based architectures to simplify circuit design and enhance the robustness of logic operations [137,138]. In this context, memristor array architectures based on majority gate (MAJ) logic have shown great potential. As a fundamental logic primitive, the majority gate can efficiently perform “sum” and “carry” computations through simple 3-input combinational logic. This approach not only fully exploits the high integration density of memristor arrays but also promotes parallel and scalable in-memory logic computing [139,140]. To illustrate this concept, the majority logic gate based on HfO*_x_* memristors (Fig. 6A) demonstrates how in-memory computing can be achieved through compact and parallel logic operations within memristor arrays [141]. The output of the majority gate is “1” if and only if at least 2 of the 3 inputs are “1”. This design combines the bipolar resistance switching characteristics of memristors and introduces an embedded series resistor R_S_ in the cross-array. Using the memristor cross-array based on the MAJ gate (Fig. 6B), they designed a parallel prefix adder (PPA). This circuit contains 7 memristor units, and by controlling the input voltage and resistance states, the operation is completed in 5 MAJ logic steps. Compared to traditional FAs, the FA implemented through the MAJ gate combination designed with memristors achieves a 4.5 times improvement in space–time efficiency. This work exemplifies the potential of majority logic in constructing highly efficient arithmetic units within memristor-based arrays, offering a promising route for compact, parallel, and energy-efficient in-memory computing architectures.

Building upon the principles of majority gate logic, memristor arrays offer a viable platform for implementing threshold logic circuits capable of handling more complex arithmetic functions [142,143]. A memristor-based crossbar architecture was employed to realize a programmable threshold logic, successfully executing both an FA and a 4-bit ripple carry adder (RCA) (Fig. 6C). The FA design leverages the differential resistance characteristics of memristors, executing logic computation through a combination of positive and negative weights alongside threshold-based comparisons [144]. Specifically, the carry-out (Cout) and sum (Sum) functions are realized via 3-input and 4-input threshold logic gates, respectively, using a memristor crossbar array. The study highlights that precise tuning of the weights and thresholds is crucial for ensuring logic correctness. With optimized configurations, the design achieved 100% accuracy in logic operations. Furthermore, a 1-bit FA was extended to construct a 4-bit RCA (Fig. 6D), which successfully computed the binary addition task “1010 + 0110 + 1”. Notably, this design circumvents endurance degradation typically caused by frequent memristor switching by utilizing read-based logic execution. Compared to traditional stateful logic implementations, it significantly reduces computational steps. This work demonstrates the feasibility of threshold logic gates in constructing accurate and energy-efficient arithmetic units in memristor-based systems, offering a scalable approach for future in-memory computing architectures.

Beyond majority gates and threshold logic, the architecture of memristor arrays can be further leveraged to implement recirculated logic operation schemes (RLOS), enabling support for more sophisticated computational applications [145]. Many cellular automata (CA) studies are implemented using a combination of CMOS circuits and FPGAs (field-programmable gate arrays), but this approach lacks flexibility [146]. The incorporation of memristors can optimize this issue. The RLOS scheme utilizes memristors to store states and perform logical operations, thereby enabling efficient computation of CA (Fig. 6E) [147]. In this design, each CA unit consists of a memristor and a transistor, where the memristor is used to store the current unit’s state and the inverted state of adjacent units, while the transistor isolates the computation to avoid cross-talk. This architecture not only eliminates the need for data movement but also significantly reduces hardware complexity. For example, the implementation based on Rule 110 (Fig. 6F) demonstrates that the method can complete complex state updates with just 3 basic logical operations. Compared to traditional methods, this approach significantly reduces hardware costs while exhibiting excellent parallel computing capabilities. This research further broadens the application scope of memristors in logic computation.

Memristors, with their unique resistive switching characteristics, offer innovative implementation paths for logic computation, evolving from majority gate logic to programmable threshold logic and recirculated logic operations. These approaches not only simplify circuit design but also provide reliable, low-cost solutions for high-performance logic computing. From basic logic gates like XOR to composable arithmetic modules such as half-adders and FAs, memristors exhibit strong scalability for constructing more complex Boolean functions. Their compatibility with crossbar architectures further enhances their ability to implement large-scale parallel logic matrices with high processing efficiency. Additionally, memristors can be configured into perceptron-like structures, integrating logic processing with neural computation and thereby unlocking significant potential for neuromorphic hardware in next-generation intelligent chips.

## Applications

With the rapid development of information technology, data security and computational efficiency have become key issues that cannot be ignored in the design of modern electronic systems [148]. Although traditional computing architectures and encryption methods have been widely used, they face increasing challenges as processing demands continue to grow [149,150]. Therefore, ensuring efficient computation while protecting data confidentiality has become a significant challenge. In this context, memristor-based logic gates offer a novel solution [151,152].

**Fig. 7.**
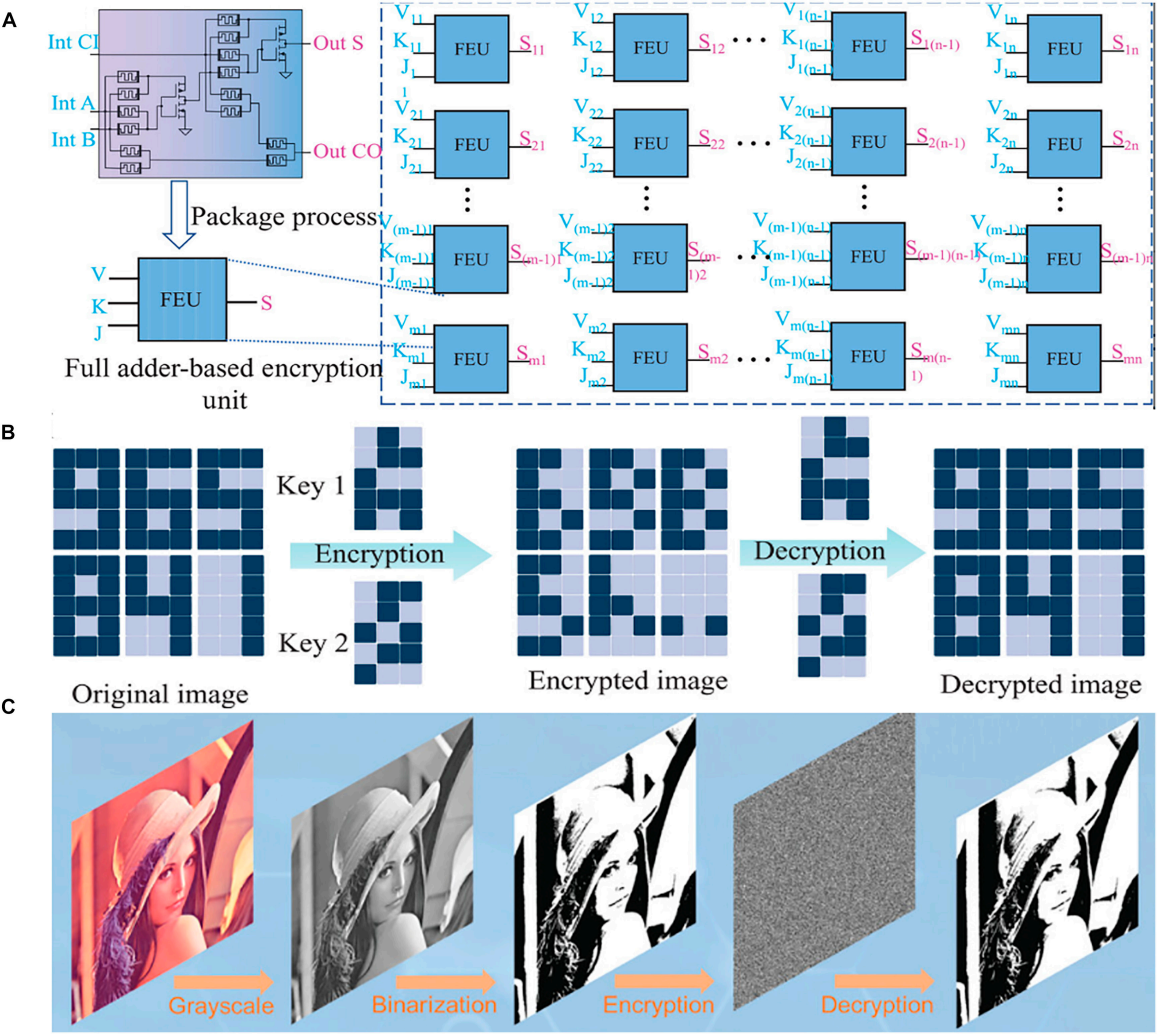
(A) Schematic representation of the FA encryption cell matrix. (B) Process of encryption and decryption for digital strings [43]. Copyright 2024, Wiley-VCH GmbH. (C) Encryption and decryption process of binarized images [133]. Copyright 2022, Elsevier Ltd.

To meet the dual demands of computation and data security, recent studies have incorporated arithmetic logic such as FAs into memristor-based encryption architectures. A hardware encryption and decryption circuit was developed using a memristor FA, which performs bit-level operations for digital strings and image data, enabling logic-based secure processing (Fig. 7A). This design leverages the logic computation capabilities of memristors, extending the FA logic function to serve as the core operation for data encryption and decryption. In the FA design shown in Fig. 7A, the input signal V corresponds to the original data to be input, while the input signals K and J correspond to the key inputs Key1 and Key2 [43]. The encryption and decryption operations are performed through the Sum output of the FA, with the decryption process following the same steps. For example, Fig. 7B shows the encryption of the digital string “965841”. The input data are converted into a binary matrix, which is then input bit by bit into the corresponding positions in the FA array. It is then processed through XOR logic operations with 2 sets of random key matrices to generate the ciphertext. The decryption follows the same FA-based procedure in reverse, gradually recovering the original data from the ciphertext. This encryption–decryption scheme demonstrates their applicability in integrated logic-memory systems that support both computation and lightweight cryptographic operations.

Using the same method, image encryption and decryption can also be achieved. An FA circuit for image encryption and decryption based on a memristor array enhances data security during the data transmission process. Figure 7C shows the image encryption and decryption operation implemented using the memristor-based FA [133]. First, the grayscale image is converted into a binarized image and represented as a binary matrix. Then, the binary matrix undergoes an encryption operation by performing logic operations with 2 sets of randomly generated key matrices using memristors. During the decryption process, the encrypted matrix is again processed with the same key matrices through FA logic operations, successfully restoring the original image. This method ensures data integrity while avoiding the potential risks of decryption associated with traditional encryption techniques. Therefore, leveraging the low power consumption and high integration capability of memristors, along with their water-soluble properties, the encryption system enables efficient hardware-level data protection, offering a practical solution for secure image transmission.

## Outlook and Challenges

**Fig. 8.**
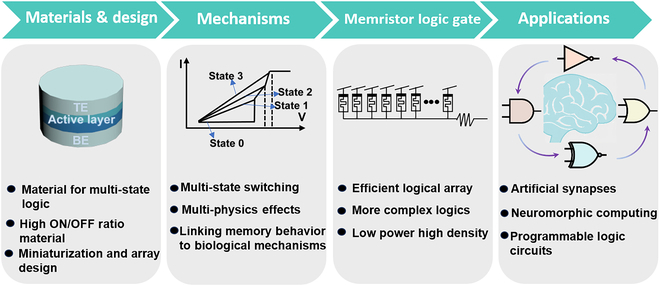
Challenges and the prospects of the memristor and logic gate. In the Materials and design section, it highlights material for multi-state logic, materials with high ON/OFF ratio, and miniaturization and array design. The mechanisms focus on multi-state switching, multi-physics effects, and linking memory behavior to biological mechanisms. It also shows the memristor logic gate, emphasizing efficient logical arrays, pursing more complex logics, and low power, high density. Finally, in the Applications, the diagram points out potential uses of memristor logic gates in artificial synapses, neuromorphic computing, and programmable logic circuits.

In summary, leveraging the unique resistive switching characteristics of memristors, researchers have explored and validated various innovative approaches to implementing logic gate functions. The core advantage of these devices lies in their ability to perform logic operations through intrinsic state transitions, inherently offering significant potential for power efficiency beyond that of traditional silicon-based devices. Combinational logic gates constructed using memristor units have demonstrated promising improvements in integration density and energy efficiency for specific applications. However, to achieve the practical deployment and large-scale integration of memristor-based logic gates, several critical challenges must still be addressed (Fig. 8). This includes the need to develop advanced materials and device structures that offer higher ON/OFF ratios, which are vital for ensuring reliable logic state discrimination and robust noise immunity. In parallel, achieving stable and tunable multi-state switching behavior is necessary to increase computational density and to support the implementation of complex multi-valued logic operations. Furthermore, it is imperative to address fabrication-related obstacles such as limited process scalability, device variability, signal interference, and interconnection reliability, all of which are essential to building high-yield and dependable memristor logic arrays.

To reliably implement and standardize multistate logic gates, it is essential to develop memristor devices with well-defined, stable, and reproducible intermediate resistance states. This can be achieved through precise control of material composition and device structure. Establishing standardized resistance intervals corresponding to each logic level is crucial for ensuring consistency across different devices and platforms. As the scale of memristor arrays increases, performance variations, noise, and parasitic effects between devices will significantly impact the accuracy of logic operations. To address these challenges, circuit-level fault-tolerant mechanisms, error correction methods, and variability-aware logic design strategies should be introduced. For example, redundancy coding, differential readout schemes, or adaptive threshold mechanisms can be employed to mitigate the influence of non-idealities. Additionally, integrating memristor arrays with CMOS control units or neuromorphic architectures may provide dynamic compensation mechanisms to enhance system robustness.

Although significant challenges remain in achieving reliable multistate logic and maintaining accuracy in large-scale memristor arrays, memristor-based logic gates provide a promising solution beyond conventional computing architectures. Their inherent advantages, such as low power consumption, nonvolatility, and the ability to perform logic operations through resistive state changes, make them suitable candidates for next-generation logic systems. Future research should focus not only on improving device-level metrics but also on redefining computing frameworks. The development of memristor logic is gradually shifting from basic logic operations to applications in neuromorphic computing, multimodal data processing, and hardware-level encryption. By integrating advances in flexible electronics, AI hardware, and 3-dimensional packaging, memristor logic gates are expected to support scalable, reconfigurable computing in edge intelligence and in-memory processing systems, paving the way for practical deployments in the post-Moore era.

## Data Availability

Data will be made available on request.
